# Determinants of fatigue in patients with Marfan syndrome: a study using PROMS

**DOI:** 10.1186/s13023-025-03880-4

**Published:** 2025-07-11

**Authors:** Ines Cavalier, Leslie Toko Kamga, Laurence Morin, Laurence Bal, Laurence Faivre, Guillaume Jondeau, Viet-Thi Tran, Olivier Milleron

**Affiliations:** 1 Department of Epidemiology, Biostatistics and Clinical Research, Hôpital Bichat- Claude Bernard, INSERM CIC-EC 1425, AP-HP.Nord-Université Paris-Cité, Paris, France; 2 Centre d’Épidémiologie Clinique, Hôpital Hôtel-Dieu, Assistance Publique-Hôpitaux de Paris (AP-HP), Paris, France; 3 Association Marfans, 121 rue de la Convention, Paris, 75018 France; 4 CRMR Marfan et Apparentés, Centre Aorte Timone, CHU Timone, Aix–Marseille Université , 264 Rue Saint-Pierre , Marseille Cedex 5, 13385 France; 5 Centre de Génétique et Centre de Référence Anomalies du Développement et Syndromes Malformatifs FHU TRANSLAD, INSERM UMR1231 Hôpital d’Enfants CHU Dijon, 14 Rue Paul Gaffarel, Dijon, 21000 France; 6 Centre national de référence pour le syndrome de Marfan et pathologies apparentés Assistance Publique Hôpitaux de Paris, Service de Cardiologie, Hôpital Bichat Paris, 264 Rue Saint-Pierre, Paris, France; 7 Laboratory for Vascular Translational Science Université Paris Cité, INSERM U1148, Paris, France; 8 Université Paris Cité, METHODS Team, CRESS, INSERM, INRAe, Paris, France

## Abstract

**Background:**

Fatigue is often reported by individuals with Marfan syndrome (MFS). However, the determinants of fatigue and its impact on the daily lives of patients with MFS remain poorly understood. We sought to assess the level of fatigue and its determinants in individuals with MFS.

**Methods:**

We conducted a cross-sectional study in ComPaRe Marfan, an e-cohort of MFS patients. Fatigue was assessed using the FACIT questionnaire already used in a wide range of chronic diseases. Pain and its interference with daily life were assessed using the Brief Pain Inventory. We performed univariate and multivariable linear regressions to identify determinants of fatigue.

**Results:**

A total of 162 people with MFS completed the FACIT-Fatigue questionnaire. The median age was 46 and 59% were women. The median FACIT-Fatigue score was 31(IQR 22–39) and over ¾ of the cohort had a FACIT-Fatigue score below 40 corresponding to at least some level of fatigue. In the multivariate model, only pain interference was associated with fatigue (Coeff= -0.34, CI95%: [-0.46; -0.22], *p* < 0.001). Beta-blocker treatment, history of aortic, lens or scoliosis surgery were not associated with FACIT fatigue score. In addition, the FACIT fatigue scores was repeated every month during 3 months and were stable over time.

**Conclusion:**

Fatigue is common in patients with MFS and is associated with pain that interferes with daily life. Therefore, pain management in MFS patients could improve quality of life and fatigue.

## Introduction

Marfan syndrome (MFS) is an inheritable connective tissue disorder mainly associated with cardiovascular, musculoskeletal, and ocular symptoms [1].

The main risk is the occurrence of aortic dissection, a potentially fatal complication of thoracic aneurysm. Thanks to advances in diagnosis, imaging techniques, genetics, cardiac surgery and beta-blockers use, over the past 50 years, the life expectancy of patients diagnosed with MFS has improved considerably, from 40 years to that of the general population [2, 3]. As a result, quality of life is an increasingly important issue. While several studies have described fatigue as a frequent and undertreated symptom, most were cross-sectional and did not investigate how this symptom varied across time [4]. Here, we aim to described the level, temporal variations and determinants of fatigue in patients with MFS.

## Methods

Participants were recruited in the Community of Patients for Research (ComPaRe), a nationwide cohort of patients with chronic conditions where a specific cohort of patients with MFS was launched in March 2020. All patients with MFS completed the Functional Assessment of Chronic Illness Therapy– Fatigue (FACIT-F) questionnaire three times at one-month intervals (M0, M1 and M2).

The FACIT-F questionnaire has been validated for cancer, other chronic diseases and the general population [5]. Its score ranges from 0 to 52, with higher scores indicating better health (i.e. less fatigue). The MYMOP is a brief, patient-developed questionnaire that asks respondents to specify the two symptoms that concern them most, and the daily activities that these symptoms limit or prevent.

We described patients’ responses to the FACIT-F and the proportion of patients reporting that fatigue was among their most important symptoms in the MYMOP-2, at M0. We assessed the factors associated with higher FACIT-F scores using multivariable logistic regressions including demographic, clinical characteristics, and pain assessments using the Brief Pain Inventory score. We assessed the temporal variation of fatigue levels in MFS by evaluating the agreement between FACIT-F scores at M0, M1 and M2 using the intraclass correlation coefficient (ICC) for agreement. All statistical analyses were performed using R version 4.2.2 (The R Foundation for Statistical Computing, www.R-project.org/).

## Results

Between July 21, 2022 and October 10, 2022, the 162 participants in the ComPaRe cohort who had completed the FACIT fatigue questionnaire were included in the study. Median age was 46 (interquartile range (IQR) 36 to 54), 96 (59%) were women and 113 reported carrying a pathogenic variant among whom 102/113 (90%) in the FBN1 gene. About 25% participants reported having comorbidities, the most frequent being cardiovascular diseases (11/162, 6.8%), respiratory conditions (7/162, 4.3%) and diabetes (5/162, 3.1%). History of lens surgery was reported in 38/162 (23.4%), scoliosis surgery in 15/162 (9.2%) and about half participants (83/162) reported a history of aortic surgery, among whom about 40% (32/83) had had an aortic dissection. Regarding treatments, 126 /162 (78%) individuals reported taking beta-blocker treatment and 11/162 (6.8%) angiotensin receptor blockers.

Median FACIT-F score was 31 at baseline (IQR 22–39) and 77.2% of the cohort had a FACIT-Fatigue score below 40 corresponding to at least some level of fatigue. Furthermore, 35/162 (22%) participants reported fatigue was among their most important symptoms and 60/162 (37%) participants reported that they strongly agreed with the item: “*I am frustrated by being too tired to do the things I want to do*”. Use of beta-blocker treatment or history of surgery was not associated with higher fatigue, at the difference of pain that interfered with daily activities (coefficient: -0.34, 95% confidence interval (CI) -0.46 to -0.22 FACIT-F score point per increase in BPI pain interference point) (Table 1).

**Table 1:** Multivariable linear regression between FACIT-F scores (at M0) and patients characteristics (*n* = 162) Positive coefficients indicate an increase in FACIT-F scores (i.e., less fatigue) while negative coefficients indicate a decrease in FACIT-F scores (i.e., more fatigue)

Characteristic	Coefficient [95% CI]	*P*-value
Age (per year)	0.03 [-0.09 – 0.16]	0.6
Male sex	0.86 [-2.33 – 4.06]	0.6
Treatment by beta blockers vs. no	-0.83 [-4.86 – 3.19]	0.68
Number of treatments for MFS (per treatment)	-0.56 [-1.14 – 0.03]	0.06
High education degree	-2.90 [-6.84 – 1.04]	0.15
Presence of a comorbidity	1.51 [-3.63 – 6.65]	0.56
BPI General pain score (per point)	-0.15 [-1.01 – 0.72]	0.74
BPI Pain Interference score (per point)	-0.34 [-0.46 – -0.21]	< 0.001

At M1, 131/162 (80.9%) patients with Marfan syndrome repeated FACIT-Fatigue questionnaire and 119/162 (73.4%) at M2. FACIT fatigue scores were stable over the three measurements (score at M1: 31 (IQR 22–40; score at M2: 32 (IQR 23–41)) highlighting no increase nor decrease of fatigue levels over time (ICC for agreement between the three measures of 0.73, 95% CI 0.66 to 0.80).

## Discussion

About 75% of patients with MFS experience fatigue (FACIT-F score < 40), 22% report that it is among their most important symptoms and 37% report being too tired to do the things they want. For comparison, the mean FACIT-F score of MFS patients in our study is 31 while it was 47 in 1,010 individuals from the US general population with a mean age of 45.7 years [6] and 43 in a large German cohort of 2426 healthy participants with a mean age of 49 years [7]. In addition, patients with MFS reported more fatigue than patients with cancer, stroke, and HIV (with mean scores of FACIT-F of 36, 38 and 34 respectively vs. 31 for MFS in this study) [5]. Our results also emphasize that fatigue in MFS is a permanent symptom, with little improvement over time. This is important new finding, as previous papers on fatigue are mainly cross-sectional and based on a single questionnaire with a low response rate. In addition, our results are consistent with previous single-center studies that used fatigue has primary outcome and found a relationship between fatigue levels and pain, but not with beta-blocker treatment [8, 9].

This study is limited by the fact that participants may not be representative of the full disease spectrum as they were predominantly female, young, highly educated, and overall more severe with rates of aortic dissection and preventive aortic surgery rates of 21% and 54% compared with 9% and 20% respectively in our overall clinical cohort [10]. However, in this MFS population from all over France, included in an e-cohort, we confirm that fatigue is frequent and linked to pain, and we demonstrate that the level of fatigue is stable over time.

## Conclusion

Fatigue is a prime and persistent symptom in MFS and is related with pain. Development and assessment of therapeutic strategies to improve fatigue in MFS, for example through better pain control, are required.

## Data Availability

The data that support the findings of this study are not openly available due to reasons of sensitivity and are available from the corresponding author upon reasonable request. Data are located in controlled access data storage at Assistance Publique des Hôpitaux de Paris.
